# The Efficacy of Cotrimoxazole for the Prevention of *Pneumocystis jirovecii* Pneumonia Among HIV-Exposed and Infected Children: A Systematic Review

**DOI:** 10.3390/epidemiologia6010008

**Published:** 2025-02-13

**Authors:** Anthony O. Agwu, Chinedu Ogbonnia Egwu, Albert Egwu Okorocha, Ifeanyi Enyanwuma, Cyril C. Amadi, Evaezi Okpokoro, Francis Patrick Akpabio, Chukwuemeka Ogbonnaya Aguwa, Donatus Onwu, Onyedikachi Nwokoro

**Affiliations:** 1 Department of Public Health, University of Chester, Chester CH1 4BJ, UK; 2 Medical Biochemistry Department, College of Medicine, Alex-Ekwueme Federal University Ndufu-Alike, P.M.B. 1010, Ikwo 482131, Nigeria; greatcyril1@gmail.com; 3 Physiology Department, College of Medicine, Alex-Ekwueme Federal University Ndufu-Alike, P.M.B. 1010, Ikwo 482131, Nigeria; okorochae@yahoo.com; 4 Surgery Department, Alex Ekwueme Federal University Teaching Hospital, Abakaliki 480282, Nigeria; enyanwuma@gmail.com (I.E.); kachinwokoro@gmail.com (O.N.); 5 International Research Center of Excellence, Institute of Human Virology, Abuja 900246, Nigeria; evaezio@yahoo.com; 6 Garki Hospital, Tafawa Balewa Way, Area 8, Garki, Abuja 900103, Nigeria; frank.pat@live.com; 7 Overcomer Jubilee Hospital, Umuahia 440221, Nigeria; emyaguwa@yahoo.com; 8 Orthopedics Department, Alex Ekwueme Federal University Teaching Hospital, Abakaliki 480282, Nigeria; onwudonatus@gmail.com

**Keywords:** HIV-related opportunistic infections, pneumonia, cotrimoxazole, prevention and efficacy

## Abstract

Background: HIV-related opportunistic infections like *Pneumocystis jirovecii* Pneumonia (PCP) remain a major contributor to child morbidity and mortality globally. PCP accounts for over 60% of AIDS in the first year of life and is responsible for a third of AIDS in children globally. Cotrimoxazole prophylaxis, which is an intervention directed towards tackling this burden, has not attained remarkable coverage despite advocacy towards scale-up. This work was therefore aimed at evaluating the efficacy of cotrimoxazole in the prevention of PCP among children exposed to and infected with HIV by carrying out a systematic review. Methods: Key scientific databases were searched for primary studies not older than 15 years old without language restrictions. Randomized Control Trials (RCTs) and Cohorts comparing the effectiveness of cotrimoxazole versus placebo in the prevention of PCP among children (<17 years) exposed to and infected with HIV were selected. Studies with a duration of follow-up not less than 3 months long were included. A meta-analysis was conducted on RevMan 5.3 statistical application software following data extraction, and the data quality and risk of bias were also assessed. Exactly Ten (10) studies were selected and analyzed. Findings: It was observed that cotrimoxazole had beneficial effects in terms of a reduction in mortality among HIV-exposed and infected children, as 72 fewer children in 1000 (based on an absolute 95% CI) will die as a result of cotrimoxazole compared to a placebo. Cotrimoxazole also significantly reduces hospital admissions (*p*-value of 0.008). The adverse events associated with cotrimoxazole are comparable to a placebo when co-administered with ARTS (*p* = 0.90), which did not impact adherence. Conclusion: The benefits of cotrimoxazole prophylaxis far outweigh its risks. Therefore, scaling up the intervention is recommended as a prophylactic for wider coverage, especially in resource-limited settings.

## 1. Introduction

The HIV epidemic in the 1980s heralded in a new dispensation in public health globally with the mortality and morbidity associated with it [1]. HIV has a strong association with major opportunistic infections like *Salmonella* infection, Tuberculosis, and *Pneumocystis jirovecii* Pneumonia (PCP), which complicates the morbidity and mortality associated with HIV [2]. Among the devastating impacts of HIV is its untoward burden on vulnerable groups like women and children and its association with poverty. It is estimated that 9 out of 10 children with AIDS will have a respiratory problem at some point in their lifetime living with HIV [3,4]. To address this massive burden, various policies, programs, and interventions have been developed over the years, one of which is the use of cotrimoxazole (a fixed-dose combination of Trimethoprim and Sulfamethoxazole (TMP/SMX) with synergistic effects) for the prevention of PCP among children who have been exposed to or infected with HIV.

PCP is the most recognized opportunistic infection responsible for mortality and morbidity among infants and children infected with HIV [5,6,7]. Symptoms of a respiratory infection associated with PCP are usually the early warning pointers to an HIV infection in children [4]. PCP is commonly acquired by children living with HIV in their first year of life. PCP is a community-acquired pneumonia (CAP), which is responsible for about 40% of childhood hospital admissions [8]. The prevention of opportunistic infections associated with HIV is a major aspect of public health intervention that has both individual and global benefits [9]. One major strategy for combatting the mortality and morbidity associated with PCP among children infected with and exposed to HIV is the use of cotrimoxazole [10]. Although cotrimoxazole is widely used for the treatment of respiratory infections like PCP, it is also used in the treatment of other childhood illnesses like malaria, urinary tract infections, and diarrhea [9]. Studies suggest that children tolerate cotrimoxazole more than adults, which makes it ideal for prophylaxis for PCP in children exposed to and living with HIV; hence, it is used as an adjuvant in the management of HIV-related bacterial infections as it helps to improve the growth and development of these children [10,11].

Despite these benefits highlighted above, there have been huge setbacks in scaling up the prophylactic use of cotrimoxazole for PCP and other opportunistic bacterial infections associated with HIV/AIDS. Since the WHO recommended this drug for the prevention of PCP, it has not had a widespread reach in developing countries where resources are limited [10,12]. Moreover, some studies suggest that PCP is more prevalent in developed countries, justifying the reason for guidelines for its use in areas like the USA and Europe. However, there are arguments that the prevalence is as high in developing countries as it is in developed countries, but due to the prevalence of more deadly diseases like Tuberculosis, children die before PCP can be diagnosed [9]. Cotrimoxazole prophylaxis coverage is still about 8% in low- and middle-income countries despite the high number of births of children exposed to HIV [10]. A recent systematic review shows that cotrimoxazole does not offer any clinical benefit as a prophylaxis in children who are HIV-exposed and uninfected except for malaria prevention [10]. However, the benefits of cotrimoxazole in HIV-exposed and infected children have not been explored in a systematic review before. This work therefore aimed to evaluate the efficacy of cotrimoxazole as compared to a placebo in the prevention of PCP among infants exposed to HIV and HIV-infected children by carrying out a systematic review in the context of mortality, hospital admission, *Pneumocystis jirovecii* isolation, adherence, and adverse events. To do this, we developed the research question “What is the evidence for using cotrimoxazole as a prophylactic agent in the management of PCP among HIV-exposed infants and infected children?”.

## 2. Materials and Methods

### 2.1. Research Design

This research was a systematic review. The following databases were used for the search: CINAHL Plus with full text, PubMed, Medline, Bio-med Central, Database of Abstracts of Review of Effects (DARE), Cochrane Library, HTA, and LILACS. The search period for all the databases was between 1 January 1991 and 28 August 2016. This period was chosen to capture the pre- and post-period of recommendation of cotrimoxazole prophylaxis in HIV infection by the World Health Organization in 2005. A ‘no language restriction’ option was maintained while searching the CINAHL database in which both the subject headings and free text terms were applied and the full-text application and abstracts were relevant. These principles were practicable and applied to other databases. However, the search was initially restricted to studies with RCTs and then extended to include cohorts due to the dearth of RCTs on the subject.

The “PICO” concept was applied to aid the choice of keywords throughout the search strategy, such as “HIV” in “exposed infants”/“infected children” as population, “cotrimoxazole” as intervention, “placebo” as comparator, and mortality/adverse effects as outcomes. The Boolean Operator “AND” was used to combine the keywords.

**Figure 1:**
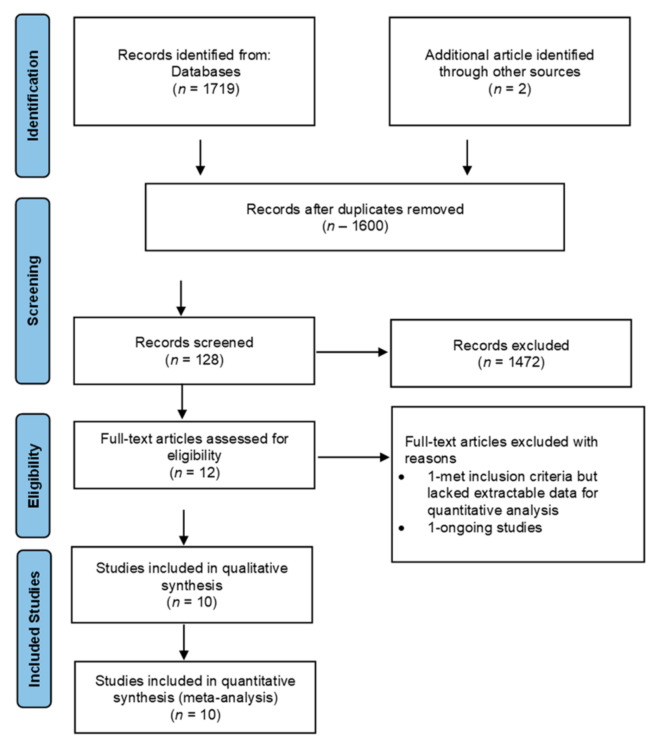
Study selection diagram: Preferred Reporting Items for Systematic Reviews and Meta-Analyses (PRISMA flow chart). The figure shows the study selection processes, including the study selection from the main databases.

The following keyword phrases were used to inform the literature search for studies: “HIV in infected children/exposed infants”, “cotrimoxazole prophylaxis”, “HIV children”, “cotrimoxazole prophylaxis”, and “PCP” AND “placebo”. The reference lists from relevant articles were examined. Data quality and risk of bias assessment were also all assessed. Ten RCTs were considered for this study, which involved 4814 participants (Figure 1). The protocol for this systematic review was registered at PROSPERO as CRD42025641554.

### 2.2. Outcome Measures

The measured outcomes for this study were as follows: mortalities related to PCP, number of hospital admissions, adverse events of cotrimoxazole, how cotrimoxazole affects adherence, and *Pneumocystis jirovecii* isolation. *Pneumocystis jirovecii* pneumonia (PCP) is a fungal infection that can be isolated in HIV patients, serving as an indicator of the disease severity. This SR assessed the effect of cotrimoxazole in reducing mortality in HIV-exposed or infected children and the number of times the patients were hospitalized. It also looked at adverse events, such as rashes, fever, and diarrhea, and the severe ones like bone marrow suppression and Steven Johnson Syndrome were also assessed in comparison to a placebo, including how these affect the level of adherence.

### 2.3. Inclusion and Exclusion Criteria

Only RCTs and Cohort studies published within the past, between 1 January 1991 and 28 August 2016, comparing cotrimoxazole and placebo as prophylaxis in the management of PCP in patients aged 6 weeks to 17 years with HIV and who were on antiretroviral therapy (ART) medications were considered. In these studies, the patients were HIV-positive and were placed on ART. Studies with duration of follow-up greater than or equal to 3 months were included. Only studies published in English Language and other languages, as long as translation was provided, were included. Case–control studies and studies where cotrimoxazole was administered for other indications were excluded. Studies involving children with other chronic conditions were also excluded.

### 2.4. Selection Process of Studies

The study selection process for this systematic review was conducted following the guidelines set out by the Cochrane Handbook of Systematic Review and the Centre for Reviews and Dissemination [13]. The initial selection process involved the review of studies by electronic search of databases, reference lists, titles, and abstracts that were regarded as relevant. This process continued with further scrutiny of the abstracts, and those that did not conform to the research question and inclusion criteria were excluded. At this stage, the selected studies were fully retrieved for further analysis. The final stage of this selection process involved more analysis of the remaining studies to be included in the systematic review in accordance with the revised Consolidate Standard of Reporting Trials (CONSORT) statement checklist [14] for their methodological quality to ensure that they were a good methodological quality fit for RCT standards. Those with poor methodological quality were omitted from the review. The selection process is shown in Figure 1.

After selecting the search strategy for each database, all studies obtained from different databases were added to EndNote X8 (Clarivate, Philadelphia, PA, USA), while the Preferred Reporting Items for Systematic Review (PRISMA) were useful in explaining how information is transcribed at various stages of a Systematic Review, as reported by Page et al. (Supplementary Material Table S1) [15]. The search was initially restricted to RCTs and then extended to include Cohorts due to the dearth of RCTs on the subject.

### 2.5. Qualitative Evaluation of Studies

In appraising the research papers, the Critical Appraisal Skills Program (CASP) (https://casp-uk.net/ (accessed on 4 October 2024)) and the New- Castle Ottawa scales (https://www.ohri.ca/programs/clinical_epidemiology/oxford.asp (accessed on 4 October 2024)) tools were deployed to evaluate the quality of the RCT and Cohort studies. To do this quality assessment objectively, the following pre-defined criteria were applied as a screening tool for each of the studies: “the appropriateness of the chosen study design in answering the research topic”, “the suitability of statistical methods used for analysis”, “the risk of bias should be assessed”, “should ascertain if the quality of intervention allocation and reporting is relevant”, and finally, “should determine if the choice of outcome measure is ideal”.

### 2.6. Data Extraction

For the data to be extracted, a ready-made checklist was used, which enquired about the first author’s name, year of publication, nature of study/duration, participants, intervention vs. comparison, and primary and secondary outcomes. This was done independently by two reviewers (AOA and COE). Any unresolved data were discussed and clarified with other authors for harmonization. The extracted data from this review were analyzed using Revman 5.3 software (Cochrane Collaboration, Vienna, Austria) as recommended by Cochrane Collaboration. Only studies with the outcome (mortality, hospital admissions, *Pneumocystis jirovecii* isolation, adherence, and adverse events) were selected. The summary of the data extraction can be found in Supplementary Material Table S2). Furthermore, GradePro 3.6, a platform/software recommended by Cochrane Collaboration, was used to grade the findings and outcomes from the analysis produced by RevMan 5.3 in terms of the quality of evidence using the GRADE (Grades of Recommendation, Assessment, Development, and Evaluation) approach (Supplementary Materials Table S3).

### 2.7. Risk of Bias Assessment

**Figure 2:**
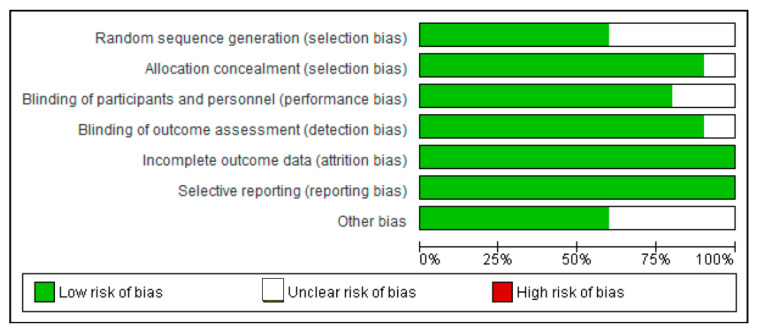
Risk of bias graph.

A risk of bias assessment was performed on each selected data using the Cochrane Collaboration tool for accessing bias to minimize the risk of bias (Figure 2).

In order to reduce potential errors, inaccuracies, and publication bias, all the steps of searching, evaluating, identifying/selecting the studies, and extracting data were performed.

### 2.8. Data Analysis

Comprehensive meta-analysis software was used to analyze the data. RevMan version 5.3 statistical tool (Cochrane Collaboration, Vienna, Austria) was deployed for data extraction with a graphical representation of the result in a funnel plot. Inconsistency index (I^2^-statistic) values of 0–40%, 30–60%, 50–90%, and 75–100% indicated unimportant, moderate, substantial, and considerable heterogeneity, respectively [13]. The Chi-square and the I^2^ statistics were used to determine the significance level and heterogeneity, respectively, while Z was used to determine the overall effect. Forest plots were constructed to demonstrate the distribution of proportion point estimates and their 95% CI for the outcomes. A funnel plot was used to show the publication bias in the article. Finally, a *p*-value set at <0.05 was used to determine the significance of the result.

## 3. Results

This work evaluated the effectiveness of cotrimoxazole in the prevention of PCP among HIV-infected and exposed children by comparing the impact of cotrimoxazole to a placebo among children living or exposed to HIV in terms of mortality, hospital admissions, *Pneumocystis jirovecii* isolation, adherence, and adverse events. RCTs and Cohorts (1719 studies were reviewed and 10 were selected) that extensively examined the efficacy of the intervention of interest were assessed manually and electronically with the assistance of different databases. After this, the exclusion and inclusion criteria for the article selection were considered using the PRISMA flow chart shown in Figure 1. This was to allow for transparency by reporting and analyzing the steps involved during the article selection process. The outcome data are represented in Table 1.

### 3.1. Mortality

**Figure 3:**
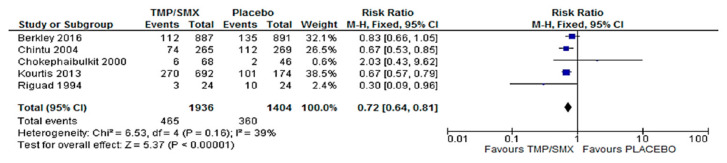
Forest plot comparing cotrimoxazole vs. placebo with mortality as an outcome; CI, confidence interval; M-H, Mantel–Haenszel; df, degree of freedom; I^2^_,_ heterogeneity; Z, overall effect; TMP/SMX, cotrimoxazole [6,12,17,19,23].

**Figure 4:**
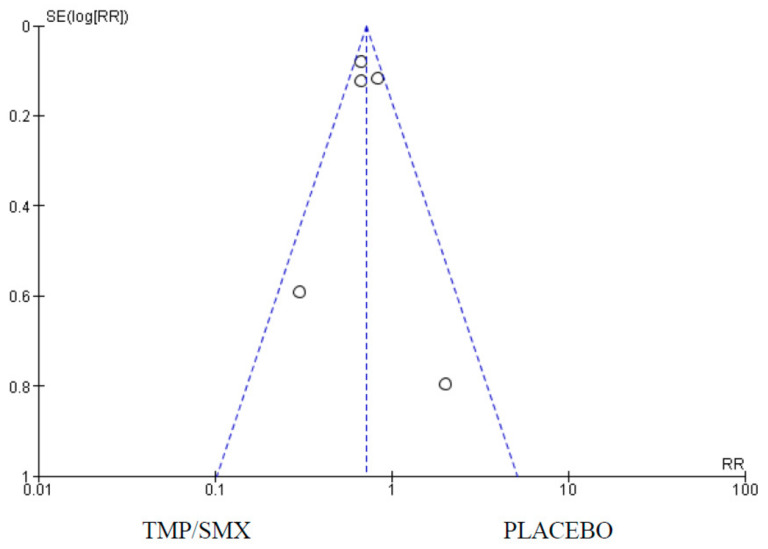
Funnel plot for comparison of mortality as an outcome between cotrimoxazole and placebo showing no publication bias; Standard Error (SE); Relative Risk (RR); TMP/SMX, cotrimoxazole. The dots on the plot represent the individual studies used.

Five (5) studies had mortality as an outcome comparing cotrimoxazole and a placebo. There were 1936 participants in the cotrimoxazole group, and 1404 for the placebo group. Forest and funnel plots were used to graphically represent the scientifically pooled results of the mortality at the end of this study (Figure 3 and Figure 4). The intervention arm of the analysis shown in Figure 3 consisted of 1936 participants using cotrimoxazole, while the control arm comprised 1404 participants treated with only a placebo. Additionally, the cotrimoxazole group displayed 465 events, while 360 events were recorded in the placebo group. The relationship between the two variables was ascertained using a chi-square test and I^2^ was used to test for heterogeneity, with the results showing Chi^2^ = 6.53 and I^2^ = 39%, respectively. Furthermore, the Z-value for the overall effect was Z = 5.37 at a *p*-value of 0.00001. RR = 0.72 (0.64, 0.81), and at a confidence interval (CI) = 95%. The publication bias or level of homogeneity in the meta-analysis is represented in Figure 4.

### 3.2. Hospital Admission

**Figure 5:**
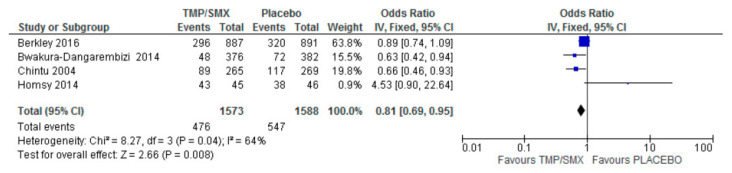
Forest plot comparing cotrimoxazole vs. placebo with hospital admissions as an outcome [12,16,18,23].

**Figure 6:**
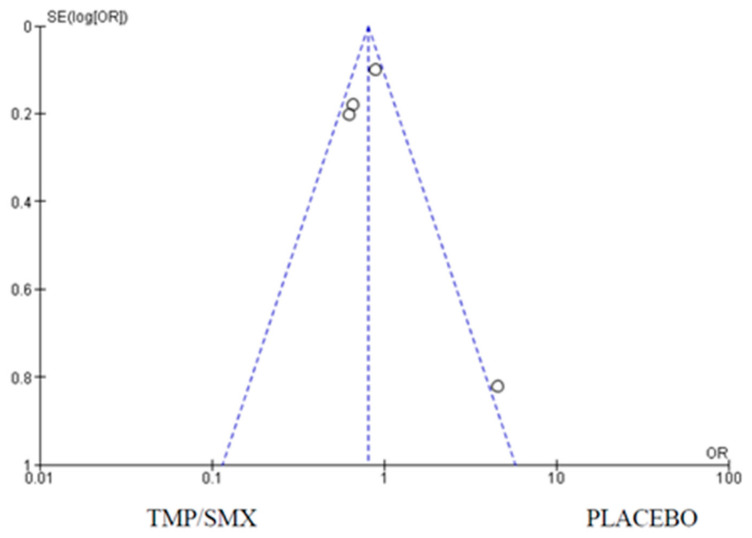
Funnel plot for hospital admissions; funnel plot for comparison of hospital admissions as an outcome between cotrimoxazole and placebo, showing minimal publication bias, as one out of the four trials is asymmetrical. The dots on the plot represent the individual studies used.

A total of four (4) studies were used to assess the impact of cotrimoxazole on hospital admissions among children infected with or exposed to HIV. Forest and funnel plots were employed to graphically represent the scientifically pooled results for hospital admission at the end of this study (Figure 5 and Figure 6). The intervention arm of the analysis shown in Figure 5 comprised 1573 participants in the cotrimoxazole group, while the control arm comprised 1588 participants treated with only a placebo. There were 476 events in the cotrimoxazole group, compared to 547 in the placebo group. The relationship between the two variables was ascertained using a chi-square test, which was Chi^2^ = 8.27, while I^2^ was employed to test for heterogeneity, which was 64%. The overall effect was Z = 2.66 and *p* = 0.008. The publication bias or level of homogeneity in the meta-analysis is represented in Figure 6.

### 3.3. Adherence

**Figure 7:**
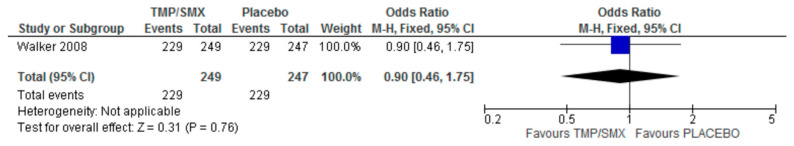
Forest plot comparing cotrimoxazole vs. placebo with adherence as an outcome [22].

**Figure 8:**
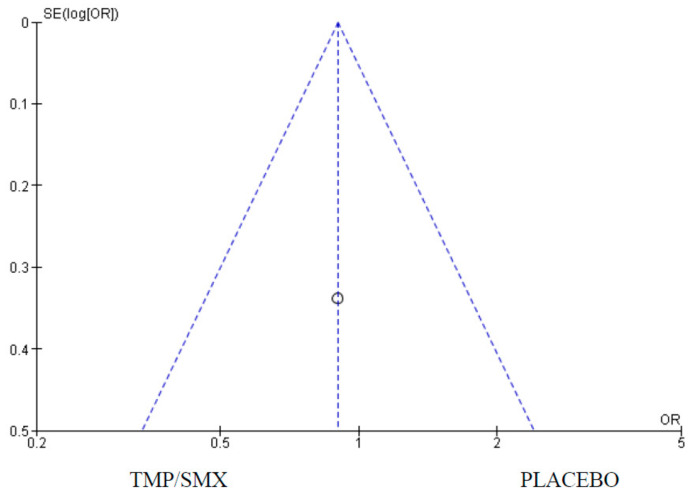
Funnel plot for adherence. The dots on the plot represent the individual studies used.

Only one (1) study measured adherence as an outcome comparing cotrimoxazole and placebo. There were 428 participants in the cotrimoxazole group and 502 in the placebo group. There were 29 events in the cotrimoxazole group compared to 85 in the placebo group (Figure 7). The publication bias or level of homogeneity in the meta-analysis is represented in Figure 8.

### 3.4. Adverse Events

**Figure 9:**
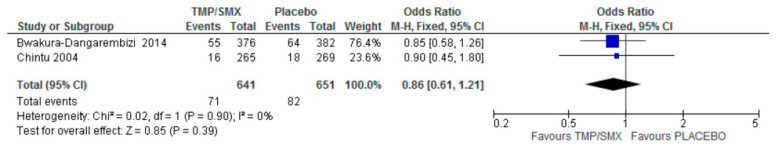
Forest plot comparing cotrimoxazole vs. placebo with adverse events as an outcome.

**Figure 10:**
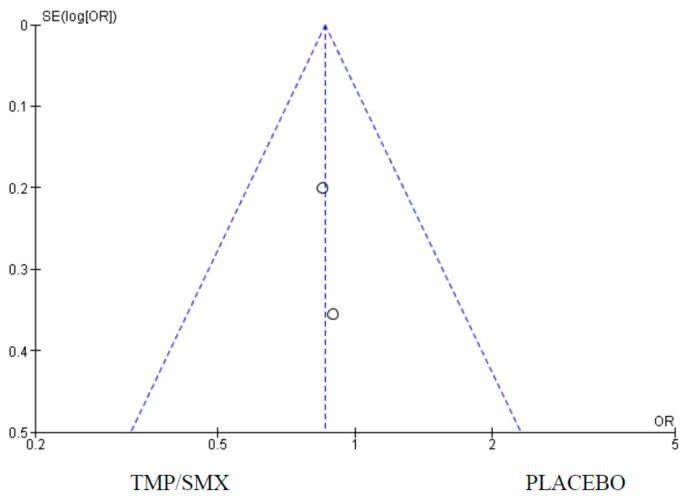
Funnel plot for adverse events; funnel Plot for comparison of adverse events as an outcome between cotrimoxazole and placebo showing no publication bias. The dots on the plot represent the individual studies used.

Only two (2) of the studies measured the difference in the number of adverse events when either cotrimoxazole or a placebo is taken with ARTs in PMCTC programs that combine interventions. There were 641 participants in the cotrimoxazole group and 651 in the placebo group. There were 71 events in the cotrimoxazole group compared to 82 in the placebo group and the statistics were as follows: Chi^2^ = 0.02, Z = 0.85 and *p* = 0.39 (Figure 9). The publication bias or level of homogeneity in the meta-analysis is represented in Figure 10.

### 3.5. Pneumocystis jirovecii Isolation

**Figure 11:**
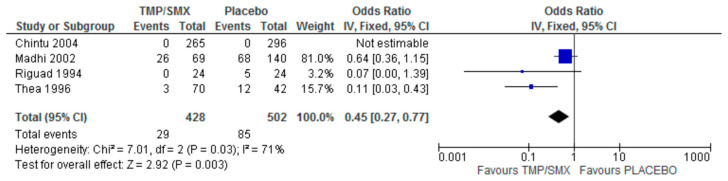
Forest plot comparing cotrimoxazole vs. placebo with *Pneumocystis jirovecii* isolation as an outcome [12,17,20,21].

**Figure 12:**
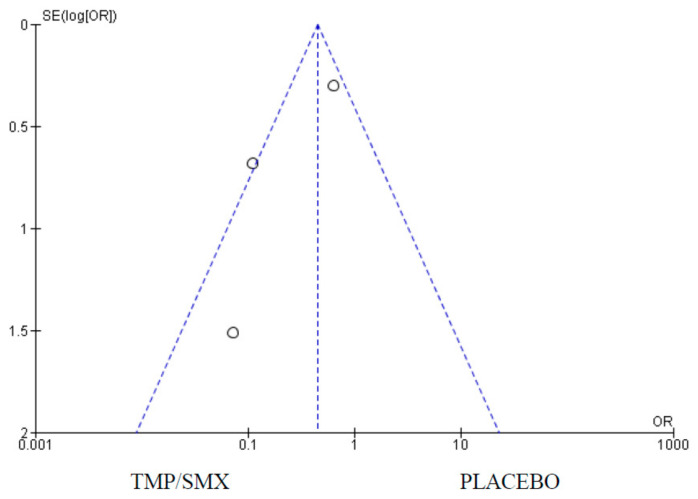
Funnel plot for *Pneumocystis jirovecii* isolation; funnel plot for comparison of *Pneumocystis jirovecii* isolation as an outcome between cotrimoxazole and placebo showing no publication bias. The dots on the plot represent the individual studies used.

In four (4) of the studies selected, the isolation of *Pneumocystis jirovecii* was a primary outcome comparing cotrimoxazole and a placebo. There were 428 participants in the cotrimoxazole group and 502 in the placebo group. There were 29 events in the cotrimoxazole group compared to 85 in the placebo group. In one of the studies, no *Pneumocystis jirovecii* was isolated in both the cotrimoxazole and placebo groups. The statistics were are follows: Chi^2^ = 7.01, Z = 2.92, and *p* = 0.003 (Figure 11). The publication bias or level of homogeneity in the meta-analysis is represented in Figure 12.

## 4. Discussion

This study was done on the premise of the need for a periodic review of global interventions for the management of HIV, such as cotrimoxazole prophylaxis, especially for the prevention of opportunistic infections (in this case, PCP) in children exposed to or infected with HIV. This drug is recommended for infants starting at 4–6 weeks after birth due to the difficulty of detecting HIV in infants [24]. This reduces the risk of getting the virus in these children born from HIV-positive mothers. The efficacy of cotrimoxazole was assessed based on its impact on mortality, hospital admissions, PCP identification, adverse events, and how it impacts adherence to ARTs in combined interventions in children.

### 4.1. Mortality

Firstly, to assess the effect of cotrimoxazole in reducing mortality in HIV-exposed or infected children, five (5) of the studies that had mortality as an outcome were synthesized. Four of the studies [12,17,19,23] favor cotrimoxazole over a placebo, except for Chokephaibulkit et al. [6]. However, the Forest plot (Figure 3) shows the overall average effect favors cotrimoxazole. The 95% CI does not cross the line of no effect. The Funnel plot (Figure 4) shows that there was no publication bias. The heterogeneity is classified as moderate. The overall (Z Test) *p*-value is less than 0.00001. This underscores the significance of this evidence. This is a critical effect in assessing the efficacy of cotrimoxazole in preventing PCP as the summary of the findings from the studies using the GRADE approach (Supplementary Material Table S3) adjudged the quality of evidence for this outcome as moderate. Seventy-two (72) fewer children in 1000 (based on an absolute 95% CI) will die as a result of cotrimoxazole compared to a placebo (Supplementary Material Table S3).

Contrary to our findings, which showed that cotrimoxazole reduces the risk of mortality, Wedderburn et al. reported in their systematic review that it does not show any clinical benefit as a prophylaxis in children who are HIV-exposed but uninfected, except to prevent malaria, which is a finding that supports those of Homsy et al. [10,18]. However, Graham et al. had previously reported that the introduction of a routine cotrimoxazole prophylaxis for infants at risk of HIV infection in several countries prevents *P. jirovecii* pneumonia, which could in itself prevent a third to a half of all HIV-related mortalities in African infants, which aligns with our findings [25]. Our finding aligns with those of Grimwade and Swinger, who conducted a review of RCTs in an area with high levels of microbial resistance to common bacteria that showed that cotrimoxazole had a 33% reduction in mortality and also had beneficial effects on hospital admissions [26]. A post-2016 review by Daniels also supports our findings as cotrimoxazole reduces mortality and morbidity risk in HIV-exposed and uninfected children, underscoring the need for a change in the policy framework to encourage its use [27]. Such adjustments may include the need to provide a dose per body weight to provide an effective and protective concentration of cotrimoxazole in children as suggested by Pressiat et al. [28].

### 4.2. Hospital Admission

The effect of cotrimoxazole in reducing hospital admissions among HIV-exposed and uninfected children as compared to a placebo was carried out with four (4) studies from the included studies that had hospital admissions as a primary outcome. Three (3) out of the four (4) studies [12,16,23] were in favor of cotrimoxazole compared to a placebo. Homsy et al. suggest an increased number of hospital admissions among children on cotrimoxazole prophylaxis [18]. However, the overall average effect is in favor of cotrimoxazole. The 95% CI does not cross the line of no effect. The funnel plot (Figure 6) suggests a minimal publication bias as one (1) of the studies, Homsy et al. [18] was plotted just outside the funnel. This does not negate the findings as there is still symmetry in the funnel plot. The slight difference in outcome may be due to minor differences in the patients’ demographics as the study designs were largely the same. A moderate heterogeneity and a *p*-value of 0.008 imply that cotrimoxazole reduces hospital admissions in this group of patients. Bacterial infections in HIV patients are one of the leading causes of hospitalization [29]; hence, the treatment of opportunistic infections can reduce hospitalizations.

### 4.3. Adherence

We assessed the effectiveness of cotrimoxazole prophylaxis in improving or promoting adherence to treatment, especially ARTs when used in combined intervention programs. Only one study, Walker et al., 2009 [22], met the inclusion criteria for this outcome. The results showed that there was no significant difference in adherence in the group that had cotrimoxazole compared with those that did not. More research and RCTs measuring this outcome could have a significant effect on this outcome.

### 4.4. Adverse Events

Lastly, it was assessed whether there would be increased adverse events in children who had cotrimoxazole compared with a placebo. Only two (2) of the selected studies that met the inclusion criteria measured adverse events as a primary outcome. The Forest plot (Figure 11) favors cotrimoxazole. However, the 95% CI crosses the line of no effect and the *p*-value is 0.90, suggesting the effect is not statistically significant. What this implies in practice is that cotrimoxazole does not increase the chance of an adverse event in children exposed to or infected with HIV who are taking ARTs. This finding is in line with the WHO’s classification of cotrimoxazole as a safe medication [10].

### 4.5. Pneumocystis jirovecii Isolation

The impact of cotrimoxazole on *Pneumocystis jirovecii* isolation was assessed with the analysis of four (4) studies. Of the four (4) studies selected, only one was an RCT, highlighting the need for more RCTs in this area. Additionally, *Pneumocystis jirovecii* was isolated in the Chintu et al. (2004) [12], study; hence, it has no impact on the overall effect. The three (3) observational studies are, however, in favor of cotrimoxazole. The 95% CI does not cross the line of no effect. The funnel plot (Figure 8) suggests no publication bias. The heterogeneity is classified as substantial. The 95% CI does not cross the line of no effect and the test for an overall effect is statistically significant with a *p*-value of 0.003, as shown in the Forest plot (Figure 7). The scarcity of data in this context calls for more RCTs.

### 4.6. Limitations of the Systematic Review

Despite the fact that the overall results showed some similarities, not all the included studies actually measured the stated outcomes. Additionally, there was a limited number of RCTs that were relevant to the focused question, which was why cohort studies were included in this review. These factors may subject this review to publication bias.

Most importantly, the majority of the studies were carried out in developing countries where the burden of HIV lies; however, the cost-effectiveness of the diagnosis of PCP, which is by broncho-alveolar lavage, makes the management of this disease condition difficult.

In addition, PCP-related deaths could not be ascertained. Although autopsies were carried out to determine the cause of death, this was only done on a fraction of the participants. The male-to-female ratio was likewise not adhered to in some of the studies, which hinders the results from being interpreted in terms of gender.

Moreover, the review did not capture studies post-2016 because it only focused on 25 years, from 1991 to 2016, within the period of recommendation of the use of cotrimoxazole as a prophylaxis by the WHO in 2005. The work did not consider articles post-2016 as it was not part of the research design. Future works will consider articles post-2016. With respect to the paucity of studies, some outcomes were captured only in one study, for instance, adherence.

## 5. Conclusions

Our findings showed that cotrimoxazole prophylaxis significantly reduces mortality and hospital admissions among children of various age groups. Despite the poor quality of reports, it is evident that the use of cotrimoxazole resulted in a reduction in isolation of *Pneumocystis jirovecii* and as such a reduction in *Pneumocystis jirovecii* Pneumonia. Moreover, this analysis reveals that there is no significant increase in adverse events with the use of cotrimoxazole. Lastly, it suggests that cotrimoxazole prophylaxis does not negatively impact adherence. Cotrimoxazole is therefore important as a prophylaxis and its usage should be intensified especially in resource-limited countries where HIV remains a public health challenge. Cotrimoxazole should be captured in the policy framework in the management of HIV-exposed and infected children considering its benefits in this group of patients.

## Figures and Tables

**Table 1:** Study outcome data.

Study (First Author/Year)	Sample Size	Experiment/Control	Type of Study	Outcomes
Chintu 2004 [12]	541	Cotrimoxazole vs. placebo	Double-blinded RCT	Mortality; HR (95% CI) 0.57 (0.43–0.77) *p* = 0.0002 Hospital admission; HR (95% CI) 0.77 (0.62–0.95) *p* = 0.01 *Pneumocystis Jarocki* identification (molecular analysis); NIL from all 73 samples
Bwakura-Dangarembizi 2014 [16]	758	Continue cotrimoxazole (376) stop cotrimoxazole (382)	Open-label randomized parallel-gap trial	Hospitalization/deaths; HR (95% CI) 1.64 (1.14–2.37) *p* = 0.007 Hospitalization/deaths from pneumonias; HR (95% CI) 1.95 (1.15–3.32) *p* = 0.01 Grade 3 or 4 adverse effects; HR (95% CI) 1.20 (0.83–1.72) *p* = 0.33 Grade 4 adverse effects; HR (95% CI) 2.04 (0.99–4.22) *p* = 0.05
Rigaud 1994 [17]	48	Cotrimoxazole vs. no cotrimoxazole (24/24)	Retrospective cohort	PCP occurrence cotrimoxazole; HR (95% CI) 0% (0–14.29%) No cotrimoxazole; HR (95% CI) 20.8% (7.1–42.2%) *p* = 0.049 Survival after 1 year; 92% of cotrimoxazole AND 74% of no cotrimoxazole Survival after 2 years; 87% of cotrimoxazole AND 59% of no cotrimoxazole. *p* = 0.035
Homsy 2014 [18]	203	Continue cotrimoxazole vs. stop cotrimoxazole	Open- label RCT + 2 observational cohorts	Hospital admissions; 43 of 116 admissions in cotrimoxazole group AND 38 of 116 admissions in no cotrimoxazole group. Adverse effects All serious adverse events; IRR (95% CI) 0.91 (0.42–1.98). *p* = 0.81
Kourtis 2013 [19]	2250	Cotrimoxazole vs. placebo	BAN RCT	Mortality; aHR 0.48. *p* = 0.03
Madhi 2002 [20]	216	Cotrimoxazole vs. no cotrimoxazole	Prospective study	Mortality among children with PCP; HR (95 % CI) 98.6% (89.1–99.8%) Isolation of *P. jiroveci*; HR (95% CI) 36% (−15.4–64.5%)
Chokephaibulkit 2000 [6]	395	Continue cotrimoxazole vs. stop cotrimoxazole	Cohort	Mortality rate; RR 0.49 (0.10–2.34) *p* = 0.47 Incidence of pneumonia; RR 0.59 (0.25–1.41) *p* = 0.22
Thea 1996 [21]	112	Cotrimoxazole vs. no cotrimoxazole	Retrospective Cohort	Incidence of PCP; un-adjustable risk of PCP in infants not receiving cotrimoxazole; 25% (12–39) Mortality; 28 deaths associated with PCP Likelihood of early death; RR (95% CI) 2.57 (1.1–6.1)
Walker 2008 [22]	534	Cotrimoxazole (249) vs. placebo (247)	CHAP randomized placebo-controlled trial	Adherence cotrimoxazole median (mean) = 92% (84%) Placebo median (mean) = 93% (82%) Rank sum Z = −0.17 *p* = 0.94
Berkley 2016 [23]	1778	Cotrimoxazole vs. no cotrimoxazole	Multicenter double-blinded randomized placebo-controlled trial	Mortality; HR (95% CI) 0.90 (0.71–1.16) Hospital admissions; 57.1 per 100 CYO (95% CI 54.6–59.6)

## Data Availability

No new data were created, so data sharing is not applicable.

## Supplementary Materials

The following supporting information can be downloaded at https://www.mdpi.com/article/10.3390/epidemiologia6010008/s1: Table S1: PRISMA checklist; Table S2: Summary of selected studies; Table S3: Assessing quality of evidence obtained using GRADE approach.

## References

[1] WHO Why the HIV Epidemic Is Not Over. https://www.who.int/news-room/spotlight/why-the-hiv-epidemic-is-not-over.

[2] Vaillant A.A.J., Naik R. (2023). HIV-1–Associated Opportunistic Infections.

[3] Lala M.M. (2023). The ‘Pulmonary Diseases Spectrum’ in HIV Infected Children. Indian J. Tuberc..

[4] Zar H.J. (2003). Prevention of HIV-Associated Respiratory Illness in Children in Developing Countries: Potential Benefits. Int. J. Tuberc. Lung Dis.

[5] Huang L., Cattamanchi A., Davis J.L., Den Boon S., Kovacs J., Meshnick S., Miller R.F., Walzer P.D., Worodria W., Masur H. (2011). HIV-Associated Pneumocystis Pneumonia. Proc. Am. Thorac. Soc..

[6] Chokephaibulkit K., Chuachoowong R., Chotpitayasunondh T., Chearskul S., Vanprapar N., Waranawat N., Mock P., Shaffer N., Simonds R.J. (2000). Evaluating a New Strategy for Prophylaxis to Prevent Pneumocystis Carinii Pneumonia in HIV-Exposed Infants in Thailand. Bangkok Collaborative Perinatal HIV Transmission Study Group. AIDS.

[7] Shetty A.K. (2005). Perinatally Acquired HIV-1 Infection: Prevention and Evaluation of HIV-Exposed Infants. Semin. Pediatr. Infect. Dis..

[8] Zar H.J., Madhi S.A., BCh M. (2006). Childhood Pneumonia - Progress and Challenges. South African Med. J..

[9] Zachariah R., Harries A.D., Luo C., Bachman G., Graham S.M. (2007). Scaling-up Co-Trimoxazole Prophylaxis in HIV-Exposed and HIV-Infected Children in High HIV-Prevalence Countries. Lancet. Infect. Dis..

[10] Wedderburn C.J., Evans C., Slogrove A.L., Rehman A.M., Gibb D.M., Prendergast A.J., Penazzato M. (2023). Co-trimoxazole Prophylaxis for Children Who Are HIV-exposed and Uninfected: A Systematic Review. J. Int. AIDS Soc..

[11] Church J.A., Fitzgerald F., Walker A.S., Gibb D.M., Prendergast A.J. (2015). The Expanding Role of Co-Trimoxazole in Developing Countries. Lancet. Infect. Dis..

[12] Chintu C., Bhat G.J., Walker A.S., Mulenga V., Sinyinza F., Lishimpi K., Farrelly L., Kaganson N., Zumla A., Gillespie S.H. (2004). Co-Trimoxazole as Prophylaxis against Opportunistic Infections in HIV-Infected Zambian Children (CHAP): A Double-Blind Randomised Placebo-Controlled Trial. Lancet.

[13] Higgins J., Thomas J., Chandler J., Cumpston M., Li T., Page M., Welch V. Cochrane Handbook for Systematic Reviews of Interventions|Cochrane Training. https://training.cochrane.org/handbook.

[14] Lepage L., Altman D.G., Schulz K.F., Moher D., Egger M., Davidoff F., Elbourne D., Gøtzsche P.C., Lang T. (2001). The Revised CONSORT Statement for Reporting Randomized Trials: Explanation and Elaboration. Ann. Intern. Med..

[15] Page M.J., McKenzie J.E., Bossuyt P.M., Boutron I., Hoffmann T.C., Mulrow C.D., Shamseer L., Tetzlaff J.M., Akl E.A., Brennan S.E. (2021). The PRISMA 2020 Statement: An Updated Guideline for Reporting Systematic Reviews. BMJ.

[16] Bwakura-Dangarembizi M., Kendall L., Bakeera-Kitaka S., Nahirya-Ntege P., Keishanyu R., Nathoo K., Spyer M.J., Kekitiinwa A., Lutaakome J., Mhute T. (2014). A Randomized Trial of Prolonged Co-Trimoxazole in HIV-Infected Children in Africa. N. Engl. J. Med..

[17] Rigaud M., Pollack H., Leibovitz E., Kim K., Persaud D., Kaul A., Lawrence R., Di John D., Borkowsky W., Krasinski K. (1994). Efficacy of Primary Chemoprophylaxis against Pneumocystis Carinii Pneumonia during the First Year of Life in Infants Infected with Human Immunodeficiency Virus Type 1. J. Pediatr..

[18] Homsy J., Dorsey G., Arinaitwe E., Wanzira H., Kakuru A., Bigira V., Muhindo M., Kamya M.R., Sandison T.G., Tappero J.W. (2014). Protective Efficacy of Prolonged Co-Trimoxazole Prophylaxis in HIV-Exposed Children up to Age 4 Years for the Prevention of Malaria in Uganda: A Randomised Controlled Open-Label Trial. Lancet Glob. Heal..

[19] Kourtis A.P., Wiener J., Kayira D., Chasela C., Ellington S.R., Hyde L., Hosseinipour M., Van Der Horst C., Jamieson D.J. (2013). Health Outcomes of HIV-Exposed Uninfected African Infants. AIDS.

[20] Madhi S.A., Cutland C., Ismail K., O’Reilly C., Mancha A., Klugman K.P. (2002). Ineffectiveness of Trimethoprim-Sulfamethoxazole Prophylaxis and the Importance of Bacterial and Viral Coinfections in African Children with Pneumocystis Carinii Pneumonia. Clin. Infect. Dis..

[21] Thea D.M., Lambert G., Weedon J., Matheson P.B., Abrams E.J., Bamji M., Straus W.L., Thomas P.A., Krasinski K., Heagarty M. (1996). Benefit of Primary Prophylaxis before 18 Months of Age in Reducing the Incidence of Pneumocystis Carinii Pneumonia and Early Death in a Cohort of 112 Human Immunodeficiency Virus-Infected Infants. New York City Perinatal HIV Transmission Collaborative Stu. Pediatrics.

[22] Walker A.S., Ford D., Mulenga V., Thomason M.J., Nunn A., Chintu C., Gibb D.M., Bangsberg D.R. (2009). Adherence to Both Cotrimoxazole and Placebo Is Associated with Improved Survival among HIV-Infected Zambian Children. AIDS Behav..

[23] Berkley J.A., Ngari M., Thitiri J., Mwalekwa L., Timbwa M., Hamid F., Ali R., Shangala J., Mturi N., Jones K.D.J. (2016). Daily Co-Trimoxazole Prophylaxis to Prevent Mortality in Children with Complicated Severe Acute Malnutrition: A Multicentre, Double-Blind, Randomised Placebo-Controlled Trial. Lancet Glob. Heal..

[24] WHO (2006). Guidelines on Co-Trimoxazole Prophylaxis for HIV-Related Infections among Children, Adolescents and Adults: Recommendations for a Public Health Approach.

[25] Graham S.M. (2002). Prophylaxis against Pneumocystis Carinii Pneumonia for HIV-Exposed Infants in Africa. Lancet.

[26] Grimwade K., Swingler G. (2003). Cotrimoxazole Prophylaxis for Opportunistic Infections in Adults with HIV. Cochrane Database Syst. Rev..

[27] Daniels B., Kuhn L., Spooner E., Mulol H., Goga A., Feucht U., Essack S.Y., Coutsoudis A. (2022). Cotrimoxazole Guidelines for Infants Who Are HIV-Exposed but Uninfected: A Call for a Public Health and Ethics Approach to the Evidence. Lancet. Glob. Heal..

[28] Pressiat C., Mea-Assande V., Yonaba C., Treluyer J.M., Dahourou D.L., Amorissani-Folquet M., Blanche S., Eboua F., Ye D., Lui G. (2017). Suboptimal Cotrimoxazole Prophylactic Concentrations in HIV-Infected Children According to the WHO Guidelines. Br. J. Clin. Pharmacol..

[29] Ford N., Vitoria M., Penazzato M., Doherty M., Shubber Z., Meintjes G., Grinsztejn B., Eholie S., Mills E.J., Davies M.A. (2015). Causes of Hospital Admission among People Living with HIV Worldwide: A Systematic Review and Meta-Analysis. Lancet HIV.

