# Supplementary material for: The Efficacy of Cotrimoxazole for the Prevention of Pneumocystis jirovecii Pneumonia Among HIV-Exposed and Infected Children: A Systematic Review
Source: Epidemiologia (Basel). 2025 Feb 13;6(1):8. doi: 10.3390/epidemiologia6010008 (PMC11843829; doi:10.3390/epidemiologia6010008)
Supplement: Supplementary file 1 [file epidemiologia-06-00008-s001.zip › Table S2_ Summary of Selected Studies.pdf]

Table S2: Summary of Selected Studies

author/ year)	Sample size	Duration of study	Study type	year	Country of study	Age of participants	Health status (HIV/ Breastfeeding)	Setting	Dose of co-trimoxazole	Loss to follow up	Outcome/ measurements
Chintu 2004	541	19 months March 2001 – Jan 2003	Double blinded RCT	2004	Zambia	6 months to 5 years	HIV infected	Children Outpatient clinics and Other clinics	<5yrs – 240Mg >5yrs – 480Mg	Death 74	Mortality Pneumocystis carinii (nasogastric aspirate) Adverse events
Bwakura-Dangarembizi. 2014	758		Open- label randomized parallel-gap trial	2014	Uganda Zimbabwe	7- 17 years	HIV infected		5-14kg – 240Mg 15-30kg-480Mg >30kg – 960Mg	9	Mortality Hospitalizations Adverse events
Riguad. 1994	48	March 1989 - March 1993	Retrospective cohort	1994	New York USA	<12 Months	HIV infected	Paediatric infectious clinic	As per guideline		Mortality PCP incidence Adverse effects
Homsy. 2014	203	Aug 2007 – March 2012	Open- label RCT + 2 observational cohorts	2014	Uganda	6 weeks – 9 months	HIV exposed	Toronto district/ rural Uganda	As per guideline		Mortality Hospital admissions Adverse reactions
Berkley. 2016	1778	2009 - 2013	Multicentre double-blinded randomised placebo-controlled trial	2016	Kenya	6 months – 5 years	NON HIV infected	2 rural/2 urban hospitals	<6 mo – 120Mg 6mo – 5 years-240Mg	352	Mortality Hospital Admissions
Kourtis. 2013	2250	2004 - 2010	BAN RCT	2013	Malawi	6 – 46 Weeks	HEU	PMTCT programs at ANC	As per guideline		Mortality
Madhi 2002	216	March 2000 – Oct 2001	Prospective cohort study	2006	South Africa	6 weeks – 3.5 years	HIV-infected	Chris Hami-Baragwanat	5Mg/Kg TMP, 20Mg/Kg SMX		Mortality PCP incidence

								h Hospital Soweto			
Chokephaibulkit 2000	395	May 1996 – Dec 1997	Cohort	2000	Thailand	1 – 6 mo	HIV exposed (infected and uninfected)	Referral hospitals in Bangkok	As per guideline	10 (2.6%)	Mortality Incidence of PCP
Thea 1996	112	1986 - 1993	Retrospective Cohort	1996	USA	<1yr	HIV infected	Six metropolitan hospitals and one outpatient clinic in New York	As per guideline	4	Mortality Pneumocystis incidence
Walker 2008	534	2001 - 2003	CHAP randomized placebo-controlled trial	2008	Zambia	1 – 14 years	HIV infected		<5yrs – 240MG >5 Yrs – 480Mg	18 17 died 1 lost to follow up	Adherence
