# Supplementary material for: The Efficacy of Cotrimoxazole for the Prevention of Pneumocystis jirovecii Pneumonia Among HIV-Exposed and Infected Children: A Systematic Review
Source: Epidemiologia (Basel). 2025 Feb 13;6(1):8. doi: 10.3390/epidemiologia6010008 (PMC11843829; doi:10.3390/epidemiologia6010008)
Supplement: Supplementary file 1 [file epidemiologia-06-00008-s001.zip › Table S3_ Assessing Quality of evidence obtained using GRADE approach.pdf]

Table S3: Assessing Quality of evidence obtained using GRADE approach

| Quality assessment | | | | | | | № of patients | | Effect | | Quality | Importance |
|---|---|---|---|---|---|---|---|---|---|---|---|---|
| **№ of studies** | **Study design** | **Risk of bias** | **Inconsistency** | **Indirectness** | **Imprecision** | **Other considerations** | **TMP/SMX** | **Placebo** | **Relative (95% CI)** | **Absolute (95% CI** | | |
| **Mortality** | | | | | | | | | | | | |
| **5** | randomised trials | not serious | not serious | serious a | not serious | none | 465/1936 (24.0%) | 360/1404 (25.6%) | **RR 0.72** (0.64 to 0.81) | **72 fewer per 1,000** (from 49 fewer to 92 fewer) | ⊕⊕⊕◯ MODERATE | CRITICAL |
| Hospital Admissions | | | | | | | | | | | | |
| 4 | randomised trials | not serious | not serious | not serious | not serious | none | 476/1573 (30.3%) | 547/1588 (34.4%) | **RR 0.08** (0.69 to 0.95) | **317 fewer per 1,000** (from 17 fewer to 107 fewer) | ⊕⊕⊕⊕ HIGH | CRITICAL |
| Pneumocystis Jirovecii Isolation | | | | | | | | | | | | |
| 4 | observational studies | not serious [a] | not serious | not serious | not serious | none a | 29/428 (6.8%) | 85/502 (16.9%) | **RR 0.45** (0.27 to 0.77) | **93 fewer per 1,000** (from 39 fewer to 124 fewer) | ⊕⊕◯◯ LOW | IMPORTANT |
| Adverse effects | | | | | | | | | | | | |
| 2 | randomised trials | not serious | not serious | serious a | not serious | none | 71/641 (11.1%) | 82/651 (12.6%) | **RR 0.86** (0.61 to 1.21) | **18 fewer per 1,000** (from 26 more to 49 fewer) | ⊕⊕⊕◯ MODERATE | IMPORTANT |
| Adherence | | | | | | | | | | | | |
| 1 | randomised trials | not serious | not serious | not serious | not serious | none | 229/249 (92.0%) | 229/247 (92.7%) | **RR 0.90** (0.46 to 1.75) | **93 fewer per 1,000** (from 501 fewer to 695 more) | ⊕⊕⊕⊕ HIGH | IMPORTANT |

**CI:** Confidence interval; **RR:** Risk ratio

a. Few studies had certain risks in one or more domains
